# Dynamic Networks in Systems Medicine

**DOI:** 10.3389/fgene.2012.00185

**Published:** 2012-09-19

**Authors:** Enrico Capobianco

**Affiliations:** ^1^Center for Computational Science, University of MiamiMiami, FL, USA; ^2^Laboratory for Integrative Systems Medicine Unit, Institute of Clinical Physiology, National Research CouncilPisa, Italy

*Why do we need “networks” in systems medicine?* Maybe there is not a simple answer. Until recently, scientists from mathematics, physics, statistics, machine learning, computer science, and similar quantitative/computational disciplines were converging to bioinformatics for known reasons. A main one was to support the experimental and theoretical work of their colleagues in medicine and biology requiring specific data analysis and inference methods in applications from complex biomedical systems. Another reason was to address more rigorously problems producing only scarce data, and thus prone to insufficient information extraction and interpretation. The paradigm of “doing more with what is available” remained in equilibrium until the next generation technologies appeared to face the “omics” challenges, and soon became the elected tools for scientific discovery.

The sign that such “technological discontinuity” is transforming research and inducing shifts of paradigms at experimental, methodological, and applied levels, is testified by the impulses – if not shocks – given to the associated computational disciplines. Such non-smooth transition has created the conditions for generating a new hybrid “scientific landscape” covering a knowledge spectrum unmet before through conventional disciplines and approaches. This landscape is hard to walk because basically endless in terms of challenges. The involved complexities concern problem indeterminacy, inaccuracy of measurements, and unknown uncertainty. To be able to at least bypass such bottlenecks, new integrative inference approaches must be designed based on qualitative and quantitative components fused into possible “targets.”

*Networks* address such needs and have naturally acquired a central role in the mentioned landscape. Many types of biological networks exist, examples being gene regulatory, protein–protein interaction, metabolic, signaling ones. While such diversity has requested particular methods and inference approaches in each application context, it did not prevent from observing common features, especially at a topological level (small world, scaling laws, etc.). Biomedical systems are measured in both physical and functional terms, and often embed “targets” to be identified, visualized in their contextual environment, and recovered for further analysis. Networks are static representations of the associations (links) between biological variables (nodes). When causative associations are present, then regulatory dynamics can be inferred. Instead, when the associations can only establish the presence of “communication” without defining directionality, then interactive dynamics are represented. Node-entities such as genes or proteins and their edge-based relationships allow the simultaneous investigation of both temporal and spatial information. Spatiotemporal dynamics are usually lacking in network maps, replaced by averages taken over conditions or time points. This limitation reduces the inherent potential of networks to emphasize roles and functions of co-existing entities through their causal relationships, while monitoring the information transmission mechanisms, including phenotypic alterations, that signaling processes activate at systems’ level. Multidimensionality is crucial for establishing a dynamic network approach based on the study of coordinated spatiotemporal signaling networks and pathways activation.

For application in *systems medicine* (Auffray et al., 2009), two other factors need to be included: implementation of a “computational multiplexing strategy” (Welch et al., 2011) and translation of systems responses to the clinic. The first factor implies steps such as the integration of heterogeneous data and variables types (signals, images, omics, and clinical variables), and the application of computational tools that can model their complex relationships and transform this knowledge into prediction power. Computational inference methods (Bayesian ones, for instance) may define the nature of the “omics-clinics” integration by casting it within efficient models. The second factor is instead assigning to systems biology an active role in changing the medical paradigms, in particular by fostering predictive, personalized, and preventive developments.

*Dynamic networks* are useful for the analysis of disease processes (Barabasi et al., 2011). Rather than individual genes and proteins, pathways and “functional modules” can be used for target identification in drug development studies, biomarker discovery, disease classification, etc. In particular, “differential network analysis” (Ideker and Krogan, 2012) may be established by comparing topological architectures and modular structures in health versus disease states, or between two different disease states, often with the support of expression, genetic, and clinical information (Cabusora et al., 2005). The expected variation may reflect the distinctiveness in molecular signatures of gene expression and protein translation arising from different combinations of genetic mutations. Once such signatures are assigned to network nodes, topological and biological features may elucidate interactions and causal relationships. Targeting altered signaling networks can suggest novel “network medicine”-based (Pawson and Linding, 2008; Zanzoni et al., 2008) therapy approaches, where the focus moves from individual targets to combined/combinatorial target dynamics.

The dynamic property of networks cannot be isolated from gene expression and pathway activation. *Transcriptome inference*, particularly in the context of the spectrum of expression levels leveraged by *RNA-Seq* features (Wang et al., 2009), i.e., depth of coverage, accurate quantification, high reproducibility, increased capture power of differential values, and also identification (and diversity) of alternative-spliced isoforms (Marioni et al., 2008), can elucidate the possible causes behind changes in the organization structure assessed at network topology scale. Even if the correlation between transcriptional and network profiles is not completely predictable due to the action of control/regulation mechanisms at both levels, a wealth of information comes from several different gene-encoded variants and from post-translational modifications involved in key proteins.

Scrutinizing “transcriptome profiles” over time and coupling it with “network configuration profiles” to find possible correlated patterns has great potential together with limitations. Such coupling (or uncoupling) could be tissue-dependent, thus a variety of studies should be performed. Then, regulatory roles of “non-coding RNA” or insight on possible “gene extensions” could be key, but not much is currently known about many types of transcript structures. However, these complexities may be monitored by looking at the changes in network configurations at both module and pathway scales. Such scales are inherently applying the principle of dimensionality reduction to high-dimensional integrative networks, as the most significant (dense) information kernels can be cohesively represented (Capobianco et al., 2011).

The sort of prediction power underlying the transcriptome could be assessed following the transfer of network dynamics into new associations (aggregation of separate interactions) and dissociations (break down) of modular structures. One could qualify such changes in transient or permanent terms (for instance, the constitutive components forming the core of pathway/complexes that change dynamically due to module addition/subtraction). Intuitively, a natural quantity to monitor is the degree of participation in “network activities” by all its constituent entities, and assuming that each module has a functional (i.e., biologically relevant) value. Thus, one objective is to check how differential conditions affect the participation to modules, which in topological terms means that properties such as betweenness and vertex–vertex distances should tell how the dynamics induce a localized or global re-positioning of vertexes in the network.

Maybe there is no simple answer to why networks are pervasive in systems medicine work; however, there are many good reasons to believe that their centrality is destined to grow, making them become bright stars for years to come.
